# Editorial: Stigma and HIV Care in Low- and Middle-Income Countries (LMICs)

**DOI:** 10.3389/fpubh.2022.900590

**Published:** 2022-04-27

**Authors:** Hailay Abrha Gesesew, Lillian Mwanri, Kifle Woldemichael, Paul Ward

**Affiliations:** ^1^Research Centre for Public Health Policy, Torrens University Australia, Adelaide, SA, Australia; ^2^College of Health Sciences, Mekelle University, Mekelle, Ethiopia; ^3^Department of Epidemiology, Institute of Health, Jimma University, Jimma, Ethiopia

**Keywords:** stigma, HIV diagnosis, antiretroviral therapy linkage, retention, UNAIDS 90–90–90, low- and middle-income countries

Human immunodeficiency virus (HIV)-related stigma is a global public health issue and substantially affects low- and middle-income countries (LMICs) which are known to be the hardest hit by HIV. Despite HIV testing services and HIV treatment (antiretroviral therapy, ART) being freely available in several LMICs, HIV testing coverage is very low, early access to ART is low, and retention in care is not satisfactory. Stigma is a cross-cutting barrier to the entire HIV care continuum, which includes the following: HIV testing and counseling, access to HIV treatment, treatment adherence, retention, and virological suppression. As such, HIV-related stigma contributes to negative HIV care and treatment outcomes such as delayed HIV diagnosis, delayed HIV treatment access, poor adherence, clinical, immunological, and virological failure, and subsequently attrition. In relation to this, in 2014, UNAIDS proposed an ambitious goal called UNAIDS 90–90–90 where respectively it was expected that 90% of individuals would know their HIV status, would receive sustained ART, and through ART would have viral suppression. The goal was planned to be achieved by 2020, however, several LMICs are yet to achieve this target by far. Stigma has been consistently named as a cross-cutting factor. As such, contextual studies are needed to explore the link between stigma and HIV care and possible strategies to halt stigma. As part of this exploration, between 1 January 2021 and 31 January 2022, a special topic entitled “*Stigma and HIV Care in Low- and Middle-Income Countries (LMICs)*” was opened and a dedicated team of scholars handled the editorial work and acted as guest editors to facilitate the timely peer-review and publication of relevant manuscripts from multiple studies (1).

A total of six manuscripts were submitted of which two were rejected and four were published. The published papers included qualitative studies from Zimbabwe and Indonesia, a mixed methods study from Ghana, and a scoping review of studies in LMICs. By February 2022, the special topic achieved over 9,300 views. As an example of the broadness of the subject covered, Kanyemba et al. explored the dynamics of disclosure, coping, and treatment adherence among adolescent boys and young men; Fauk et al. explored the perspectives and personal experiences of health workers about HIV stigma and discrimination toward people with HIV; Wowolo et al. assessed the impact of different parental figures of adolescents living with HIV; and Septarini et al. reviewed how to collate existing information about stigma-related research in LMICs among men who have sex with men (MSM) using methodological frameworks.

Because the emergence of the COVID-19 pandemic took the attention of research in 2020/21, fewer than expected abstracts were submitted to this stigma-related special issue despite stigma's multipronged impacts and it being a cross-cutting barrier to the entire HIV care continuum. Nevertheless, these published articles covered different age and population groups, methodologies, countries, and perspectives. It is also important to note that this special topic reflects the continued investment of the research community, the supporting editorial team, and the Frontiers in Public Health publishing staff to facilitate the publication process in times of crisis.

## Author Contributions

HAG drafted the manuscript. All authors listed have made a substantial, direct, and intellectual contribution to the work and approved it for publication.

## Conflict of Interest

The authors declare that the research was conducted in the absence of any commercial or financial relationships that could be construed as a potential conflict of interest.

## Publisher's Note

All claims expressed in this article are solely those of the authors and do not necessarily represent those of their affiliated organizations, or those of the publisher, the editors and the reviewers. Any product that may be evaluated in this article, or claim that may be made by its manufacturer, is not guaranteed or endorsed by the publisher.
